# Protect Others to Protect Myself: A Weakness of Western Countries in the Face of Current and Future Pandemics? Psychological and Neuroscientific Perspectives

**DOI:** 10.3389/fnint.2021.608151

**Published:** 2021-04-22

**Authors:** Martial Mermillod, Tiffany Morisseau

**Affiliations:** ^1^LPNC, Univ. Grenoble Alpes, Univ. Savoie Mont Blanc, CNRS, Grenoble, France; ^2^Université de Paris, Laboratoire de Psychologie et d’Ergonomie Appliquées, Boulogne-Billancourt, France; ^3^Laboratoire de Psychologie et d’Ergonomie Appliquées, Univ. Gustave Eiffel, Versailles, France; ^4^Strane Innovation, Gif-sur-Yvette, France

**Keywords:** pandemics, cognitive neuroscience, intercultural psychology, COVID-19, social neurosciences, perspective-taking, SARS-CoV-2

## Abstract

The COVID-19 pandemic has generated a large number of publications in the medical and biological fields concerning the virus and its treatments, as well as in psychology, social sciences, and data sciences with regard to the spread of the virus. Surprisingly, far fewer neuroscientific articles have been published in this field of research and one might well ask whether the cognitive neurosciences have anything to say at all about this vital topic. In this article, we highlight a research perspective relating to differences in the individual perception of the pandemic in Western compared to Eastern countries. Although this problem is complex, multifaceted and subsumes many other social variables, we suggest that the cognitive neurosciences do have important and fundamental insights to contribute concerning the collective response observed within these populations. More precisely, we propose the hypothesis that differences in the propensity to adopt a holistic perception of contamination processes at the group level, involving brain structures that are also associated with perspective-taking and empathy such as, in particular, the medial prefrontal cortex (MPFC) and the anterior cingulate cortex (ACC), could help explain the differences in the perception of the pandemic observed between Western and Eastern countries.

## Introduction

**Figure 1:**
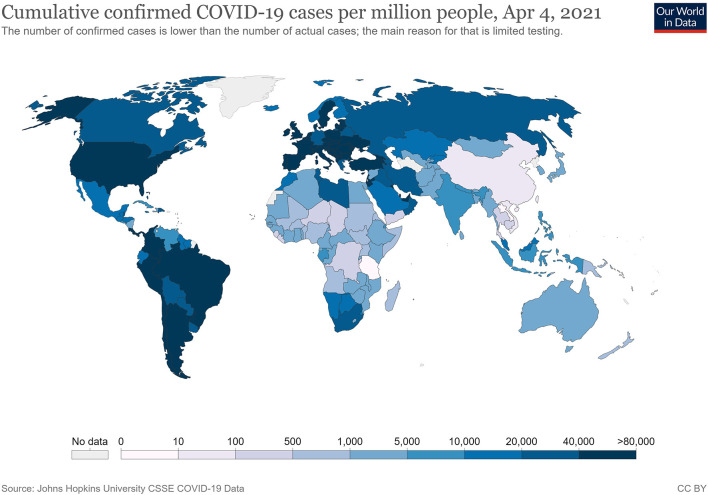
Cumulative confirmed COVID-19 cases per million people (these data could be modulated by testing efficiency). Source: Johns Hopkins University, on April 04, 2021.

**Figure 2:**
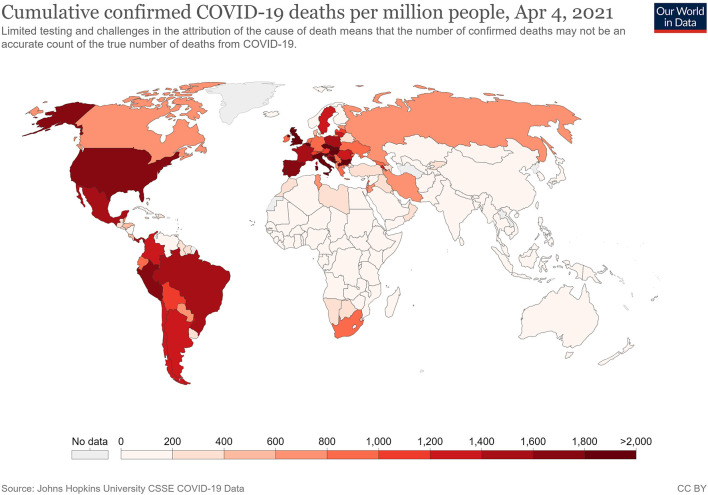
Cumulative confirmed COVID-19 deaths per million people (these data could be modulated by attribution of the cause of death). Source: Johns Hopkins University, on April 04, 2021.

All epidemiological studies show that the COVID-19 pandemic is hitting Western countries harder than Asian ones (Figures 1, 2), and that it is doing so independently of biological or regional factors (e.g., differences in the weather, et cetera) The huge differences in health performance among major Asian countries (particularly China, Japan, South Korea and Vietnam) and those of Europe and America also demand other explanations (Yamamoto and Bauer, 2020). The scale of this crisis makes it particularly important for Western countries to handle since further pandemics are highly probable, given the massive use of antibiotics in intensive farming for meat consumption, habitat destruction and exposure to vast wildlife reservoirs, among other factors (McNeely, 2021).

It has been suggested that various specific factors such as stress and coping, navigating threats, social and cultural influences on behavior or public policies have played a role in the spread of the virus (Gibney, 2020; Maier and Brockmann, 2020; Van Bavel et al., 2020a). However, and intriguingly, the difference in contamination rate between Western and Eastern countries seems to be independent of more general factors such as technological, economic or political development. For example, countries governed by populist or an authoritarian regimes like Hungary, Poland, Russia, Brazil or USA have experienced a higher contamination rate compared to China, an authoritarian regime, but also compared to South Korea or Japan, which are more liberal regimes. This has led researchers to explore other, perhaps more fundamental, factors that could have contributed to this difference. In particular, we argue that the behavioral and cognitive neurosciences can contribute to our understanding of why authorities have taken so much time to react and whether, more generally, Eastern populations were culturally ready to adopt the collective mechanisms required to reduce the spread of a virus.

We assume here that the best behavioral solution for reducing the spread of the pandemic probably lies in a collective response whose goal is the protection of society at a collective level. However, to be effective, such an approach requires individuals to adopt prosocial behaviors that, from a purely individual standpoint, might appear economically irrational. In this article, we focus on a novel and so far unaddressed hypothesis that could contribute to and possibly enhance our understanding of the observed differences between Western and Eastern countries on the basis of psychological and neurological factors. We consider the possibility that psychological and neurological differences that exist at a cultural level (Triandis, 2018) have influenced the collective capacity of countries to take appropriate actions to counter the pandemic.

## Behavioral and Psychological Differences in Collective Responses to The Pandemic

In Western countries, the dominant attitude toward, and behavioral response to, the pandemic has focused on protection at the individual level. It is from this perspective that physical distancing, hand-washing, lockdown strategies, et cetera. have been recommended by governmental advertising campaigns (Drury et al., 2021), especially at the beginning of the pandemic, and these have been understood as such by a vast majority of Western people, even though this way of framing the problem was not the most effective (Lunn et al., 2020). However, strategies based on individualistic considerations alone are not sufficient, all the more so if public health guidance is applied without a real understanding of the importance of these behaviors in preventing the contamination of others (Timmons et al., 2020). Collective restriction measures can be effective at the collective level (and thus indirectly benefit each individual in the group, all of whom prefer to live in a virus-free society). However, at the individual level, a rational actor often has an interest in benefiting from collective action without taking part in it (an instance of the free-rider paradox, in which people who benefit from a shared resource are not contributing their fair share of the costs). Rational behavior from a collective standpoint may thus no longer appear as rational from an individual perspective alone (Cato et al., 2020). Focusing only on the benefits of constraining measures in terms of protection for oneself against the virus might lead to considering such measures as excessive, especially in people who are less at risk or for whom the cost of following the rules is simply too great (Elcheroth and Drury, 2020). For instance, one of the simplest and most efficient ways to stop a virus that spreads through the respiratory tract is to wear a mask (Chu et al., 2020). However, when faced with the urgency of a rampant pandemic, authorities and individuals in Western countries took several months from the beginning of the pandemic to adopt this very simple behavior.

Studies such as that conducted by Chu et al. (2020) also indicate that wearing a mask is a limited strategy for protecting ourselves because contamination occurs by propagation of the virus through the hands, the face, or the eyes. Masks are much more efficient at protecting others given that the virus propagates outside the body, primarily through our respiratory tracts. Indeed, the effectiveness of wearing a mask as a means of individual protection is not very intuitive, particularly for populations at low risk because of their age or those living in sparsely populated areas.

The collective response became more effective as governmental communications began to focus on protecting others, by highlighting for instance the positive effects of such measures on more vulnerable people or on the sustainability of the healthcare system (Everett et al., 2020). Wearing a mask indeed becomes optimally efficient even at an individual level if everyone shares the single common goal of protecting society as a whole. As the economic and social consequences of the pandemic become more severe, the importance of recovering quickly from the pandemic at the societal level may become more evident to individuals. However, for this to happen, putting the interest of the group at the center of people’s concerns is critical.

In Eastern populations, the habit of wearing a mask already existed prior to the pandemic, generally in connection with air pollution problems but also, and more specifically, when people were sick. Interestingly with regard to our argument, this behavior is not adopted to protect the sick individual wearing the mask, but to protect their entourage. Could it be that the importance of protecting others and society as a whole has been cognitively less salient to individuals in Western countries compared to Eastern countries?

As we outlined above, the best way to deal with a pandemic consists in coordinated protective measures which require us both to consider collective rather than individual goals, including the protection of people we do not know, and also to trust others to make pro-social choices. A recent body of literature has documented how shared social identification with one’s family or community (Vignoles et al., 2021) or national identification (Van Bavel et al., 2020b) predict normative actions and support for public health measures in the context of COVID-19. Messages focused on duties and responsibilities toward family, friends and fellow citizens also seem to be an effective approach for public health messaging (Everett et al., 2020). To what extent then does collectivist (as opposed to individualistic) psychology actually facilitate efficient decision-making given that it involves choices that are *a priori* less rational for oneself, but collectively effective?

Previous behavioral and neuroscientific evidence of the difference between Eastern and Western countries has suggested that collectivist reasoning in Asia is very different to the dominant individualistic response observed in Western countries (Nisbett et al., 2001; Matsumoto and Juang, 2016; Kitayama et al., 2018; Triandis, 2018). In their review, Markus and Kitayama (2010) describe that how culture, self and their interaction shape psychological functioning. The important work of the social psychologist Shinobu Kitayama, in particular, has shown, for example, that rice-growing civilizations are more interdependent and base their reasoning more on holistic thinking than wheat-growing civilizations, which are more independent and promote individualistic behaviors (Talhelm et al., 2014). These differences have been shown to influence a wide variety of behaviors, ranging from visual perspective-taking and intentionality (Wu and Keysar, 2007) to the valuing of scarce objects (Diesendruck et al., 2019). They demonstrate how cognitive and motivational processes can be influenced by culture and could explain why individuals from collectivist societies display a kind of faith in the usefulness of collective measures that, at the individual and local level, may be perceived as irrational.

## A Neuroscientific Perspective

At the neuroscientific level, differences in inter-cultural behaviors correlate with differences in the specific neural structures involved in these behavioral responses, suggesting that long-term repetitive behaviors could be hardwired in brain structures and, in turn, produce heuristic behaviors that are difficult to suppress. Of course, stating that these types of behaviors originate in the brain could be considered as a truism. At this point of the reasoning, one could question the added value of neuroimaging studies, since experimental evidence already exists in psychology and the social sciences.

We argue that, beyond providing evidence concerning the neural underpinnings of cognitive processes, neuroimaging studies can define important constraints for the models proposed by psychology, as has been the case in the field of memory, perceptual categorization, emotional processes, etc. Moreover, they can also lead to interesting insights about how repetitive behaviors can shape the brain and how, in turn, the brain can influence our perception of the environment in the long run. In this respect, Amodio and Frith (2006) have, on the basis of their review of the literature relating social cognition to the medial frontal cortex, proposed a model of how the brain shapes our political attitudes, thereby providing invaluable insights in this emerging field of research. We suggest that integrative neurosciences, working together closely with psychology, psychophysiology and neuropsychology (Nam et al., 2021) and as a complement to the social sciences, could have a major role to play in our understanding of the multifaceted aspects of this pandemic crisis.

The cognitive neurosciences have identified these types of neurofunctional differences in holistic reasoning between Eastern and Western countries at both a functional (Kraus and Kitayama, 2019) and anatomical level, with a higher volume of gray matter in the temporoparietal junction (TPJ), an area related to this form of reasoning (Kitayama et al., 2020), being observed in the former. Additionally, evidence has been found of neurofunctional differences at the level of the medial prefrontal cortex (MPFC) and the anterior cingulate cortex (ACC) between Western and Eastern populations. These differences involve the representation of the self and could constitute an important factor explaining these types of behavioral differences (Zhu et al., 2007). The MPFC is involved in self-relative to other-judgments (e.g., Craik et al., 1999) and in the representation of self-knowledge such as one’s own personality traits, whereas the anterior cingulate cortex (ACC) is largely involved in perspective-taking and empathy (Decety, 2005). It should be noted that the results reported by Zhu et al. (2007) suggest that MPFC is activated by self- and intimate-other-judgments in Chinese individuals (i.e., implying a more collective processing of the self), whereas it is activated only by self-judgment in Westerners.

In order to test the perspective-taking hypothesis proposed in the current article, experimental designs such as the fMRI protocol used by Jackson et al. (2005) for empathy related to pain—in which participants were asked to assess levels of experienced pain based on photographs—could be applied to a specific pain related to viral contamination (affecting ourselves or others). All in all, neural perspective-taking abilities could be an important factor, modulated by the cultural context and assessed using modern cognitive neuroimaging techniques that could in turn have a major impact on the perception of the spread of the pandemic and behavioral action taken to prevent it.

Of course, such differences can be modulated *via* brain plasticity mechanisms. Neural plasticity has been widely shown to increase the volume of different brain areas related to repeated behaviors (Draganski et al., 2004). Similar neural plasticity processes can occur in relation to social behaviors, as has been shown in the case of perspective-taking abilities (Talhelm et al., 2014), as recently suggested in connection with political attitudes (Kanai et al., 2011).

This body of neuroscience studies is particularly interesting because it suggests that a particular brain network (associated with a reduction in the volume of the anterior cingulate cortex) may be more or less adapted to react collectively in an optimal way. This hypothesis is based on psychological and neuroscientific evidence that neurofunctional differences regarding the representation of the self at the level of the MPFC and the ACC between Western and Eastern populations could constitute an important factor explaining these types of behavioral differences (Zhu et al., 2007). An interesting hypothesis that could be further explored is that the lower propensity of Western countries to adopt a global rather than individual perspective in the collective response to the pandemic is further exacerbated by neurological patterns which are more oriented towards the processing of relevant information at the individual level and lead to longer delays in the implementation of efficient measures against the pandemic.

In the context of a pandemic, which requires a high degree of reactivity, deep-seated individual preferences could make it more difficult for us to favor choices that are *a priori* less rational for oneself if we do not live in a culture that normally promotes a more societal perspective. In other words, the tendency to respond in an optimal way at the collective level might not depend only on what people *know* to be best (epistemic factor) or on the behavior of others (social factor), but also on a tendency to reason at a collective level and this is only likely to change in the long run.

## Conclusion. from Pandemic to More General Perspectives

We propose that psychological differences in perspective-taking, mediated by brain networks involved in this ability (MPFC and ACC), could contribute to the differential responsiveness of Western vs. Eastern countries in the face of pandemics. We underline the importance of perspective-taking processes in the propensity to take the appropriate decisions in a pandemic situation, both at the political and individual levels, in order to stop the propagation of such a pandemic in good time. This perspective can reconcile two lines of results that might be seen as paradoxical. On the one hand, conservative and authoritarian attitudes are generally correlated with higher levels of disgust and fear of contamination (Inbar et al., 2012). On the other, anti-mask attitudes during the pandemic (including the belief that masks are ineffective or psychological resistance to being forced to wear a mask) have been linked to political conservatism (e.g., Taylor and Asmundson, 2021). Even though multifactorial issues are arguably involved in this paradox, it is interesting to note that conservative and authoritarian attitudes have been associated with smaller anterior cingulate cortex volumes in the political neuroscience literature (Kanai et al., 2011). It might be that a lower propensity to take a collective perspective when evaluating protective measures whose effectiveness lies at the societal level could fuel negative attitudes from the most conservative part of the population in countries such as Brazil or the United States.

Of course, many other factors must be considered in order to understand the root of this multifaceted effect, such as the decades-long reduction in funding of the public health systems in Western countries, the dependency of these countries in terms of strategic capacities as a result of globalization, as well as other economic, societal and psychological factors, including individual and collective obedience, level of education, etc. However, we can speculate that perspective-taking is probably involved in avoiding the contamination of others and that including this factor in statistical analyses might possibly mediate important differences between cultures and political orientations. We also stress the importance of dealing with this potential weakness of Western countries in order to respond to future pandemics that may possibly be worse than the current crisis. The most effective way to improve and orient these perspective-taking competencies towards a more holistic perception of society as a whole is probably through education. Importantly, the efficiency of educational interventions in this direction could be assessed both at a behavioral level, by evaluating perspective-taking capacities, and at a neurological level, by evaluating the neural plasticity occurring at the level of the brain structures associated with this learning and using an approach similar to that adopted by Draganski et al. (2004).

We believe that this is an interesting line of research to pursue, not least because the importance of perspective-taking and the neural structures involved in the processing of common goals could be important for other societal domains beyond the pandemic crisis. Recent studies in political neurosciences (Zmigrod and Tsakiris, 2021) stress the importance of understanding the psychological and neurological mechanisms underlying decision-making in political contexts and of better addressing the major economic, societal and ecological challenges of this century. One could indeed consider that perspective-taking and holistic social information processes have implications that go far beyond the efficiency of collectivist reasoning in the Eastern world in response to the current pandemic issue. For example, at the economic level, we would like, in future research, to consider the possibility that perspective-taking and a holistic understanding of the benefits of economic levers could be a key factor in adherence to wealth redistribution, potentially leading to global economic growth and social peace. Such a perspective may also be relevant when tackling security issues linked to the unequal distribution of wealth and ghettoization phenomena, and may help to promote collective adherence to efficient political measures that make it possible to alleviate the consequences of these processes.

In summary, we suggest that perspective-taking directed at one’s community as a whole is a key aspect of our collective capacity to face a variety of societal problems. Gaining a better understanding of the psychological mechanisms behind this tendency, together with their neurological correlates, may be an important step in building a more intelligent and resilient post-pandemic world.

## Data Availability Statement

The original contributions presented in the study are included in the article, further inquiries can be directed to the corresponding author.

## Author Contributions

MM wrote a first version of the article. TM added substantial contributions including important theoretical content to the revised version. All authors contributed to the article and approved the submitted version.

## Conflict of Interest

TM was employed by Strane Innovation. The remaining author declares that the research was conducted in the absence of any commercial or financial relationships that could be construed as a potential conflict of interest.
